# Primary testicular lymphoma: A case report highlighting 18 F-FDG PET/CT role

**DOI:** 10.1016/j.ijscr.2025.111677

**Published:** 2025-07-14

**Authors:** Raja Sfar, Meriem Ouachem, Fethi Jebali, Mohamed Ali Chaouch, Mohamed Zayati, Kawthar Chatti

**Affiliations:** a Department of Nuclear Medicine, Sahloul University Hospital, Sousse, Tunisia; b Biophysics research laboratory TIM LR12ES06: Technology and Medical Imaging of the Faculty of Medicine of Monastir, Tunisia; c Department of Anesthesiology B, Monastir Maternity Hospital, Monastir, Tunisia; d Department of Visceral and Digestive Surgery, Monastir University Hospital, Monastir, Tunisia

**Keywords:** Primary testicular lymphoma, Diffuse large B cell lymphoma, 18F-FDG PET/CT, Non-Hodgkin lymphoma, Testicular tumor, Chemotherapy, Staging, Disease progression, Case report

## Abstract

**Background:**

Primary testicular lymphoma (PTL) is a rare but aggressive form of non-Hodgkin lymphoma (NHL) that primarily affects older men. Diagnosis is challenging, and early detection is crucial for improving patient outcomes. 18F-fluorodeoxyglucose positron emission tomography/computed tomography (18F-FDG PET/CT) plays a pivotal role in the diagnosis, staging and monitoring of primary testicular lymphoma, providing detailed metabolic and anatomical information that guide therapeutic strategies.

**Case presentation:**

A 56-year-old male with no significant medical history presented persistent right testicular pain for 15 days. The initial ultrasound findings suggested presuppurative orchiepididymitis, which did not resolve with antibiotic therapy. Subsequent imaging revealed suspicious testicular architecture and inguinal lymphadenopathy. The right orchiectomy confirmed the diagnosis of diffuse large B-cell lymphoma. 18F-FDG PET/CT demonstrated widespread nodal involvement and possible bilateral testicular primary disease. The patient underwent chemotherapy with R-CHOP, but was lost to follow-up. His condition deteriorated and disease progression led to his death a few weeks later.

**Clinical discussion:**

PTL typically presents as a painless scrotal mass with a high tendency for bilateral and systemic involvement. Staging and treatment include orchiectomy, chemotherapy, and sometimes radiation therapy, although there is no standardized protocol. 18F-FDG PET/CT significantly improves diagnostic accuracy, allowing for precise staging and evaluation of treatment response. However, PTL remains a highly aggressive disease with poor overall survival and frequent relapses, often affecting the central nervous system.

**Conclusion:**

This case underscores the importance of early diagnosis and comprehensive management of PTL. 18F-FDG PET/CT serves as an invaluable tool in detecting primary and metastatic involvement, guiding treatment decisions, and monitoring disease progression. Further research is needed to optimize treatment strategies and improve patient outcomes.

## 1 Introduction

Primary testicular lymphoma (PTL) accounts for 1 to 2 % of all cases of non-Hodgkin lymphoma (NHL) and <5 % of all testicular tumors, although this proportion increases significantly with age. In men over 50 years of age, PTL is the most common form of testicular tumor [1]. Inguinal orchiectomy is the preferred diagnostic (and therapeutic) procedure, with consideration for a blind biopsy of the contralateral testicle. Treatment for primary testicular lymphoma (PTL) typically involves orchiectomy, chemotherapy, and sometimes radiation therapy, although there is no standardized treatment protocol. It is a highly aggressive neoplasm with poor rates of progression-free survival and overall survival [2]. 18F-FDG PET/CT has become an essential tool for the staging, diagnosis, and follow-up of PTL. It offers superior sensitivity and specificity compared to other conventional imaging modalities. This case report, reported according to SCARE guidelines [3], aims to allow for simultaneous assessment of metabolic activity and tissue anatomy.

## 2 Case presentation

**Fig. 1:**
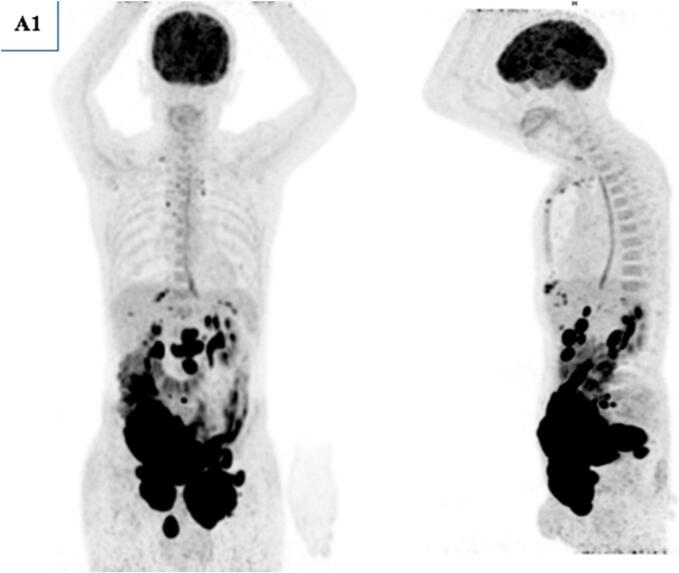
(A1) MIP (Maximum Intensity Projection) face and profile of the initial PET.

**Fig. 2:**
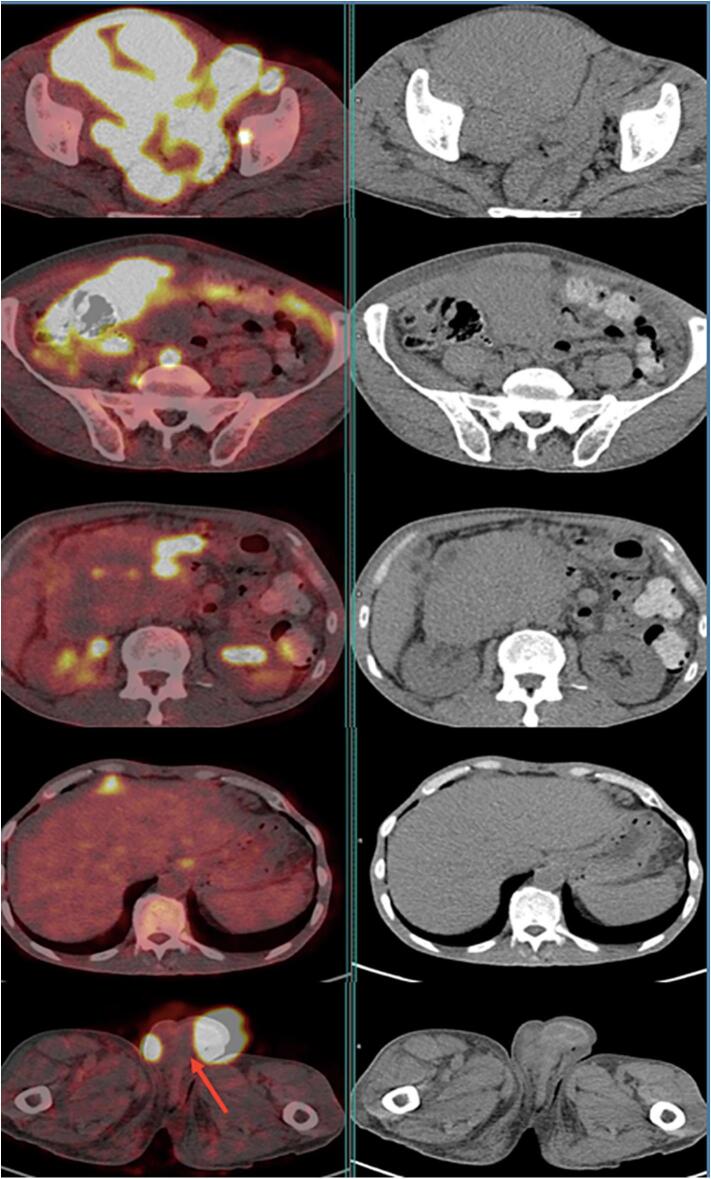
PET-CT fusion images and axial-slice CT images of the initial PET scan showing supra- and subdiaphragmatic lymph node involvement with bilateral primary peritoneal and testicular involvement (red arrow). (For interpretation of the references to colour in this figure legend, the reader is referred to the web version of this article.)

**Fig. 3:**
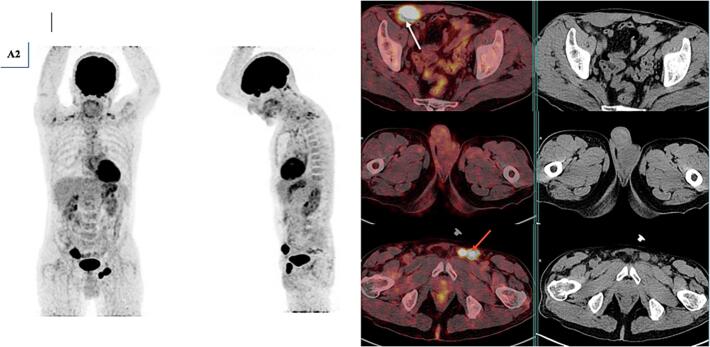
(A2) MIP (Maximum Intensity Projection) face and profile of the final treatment PET. PET-CT fusion images and axial slice CT images of the initial PET scan showing the persistence of 2 left inguinal lymph nodes (red arrow) and a hypermetabolic abdominal parietal lesion (white arrow) with good partial metabolic response (Deauville 5), and the disappearance of hypermetabolic supradiaphragmatic and testicular lymph node lesions, as well as the majority of subdiaphragmatic and peritoneal lesions. (For interpretation of the references to colour in this figure legend, the reader is referred to the web version of this article.)

**Fig. 4:**
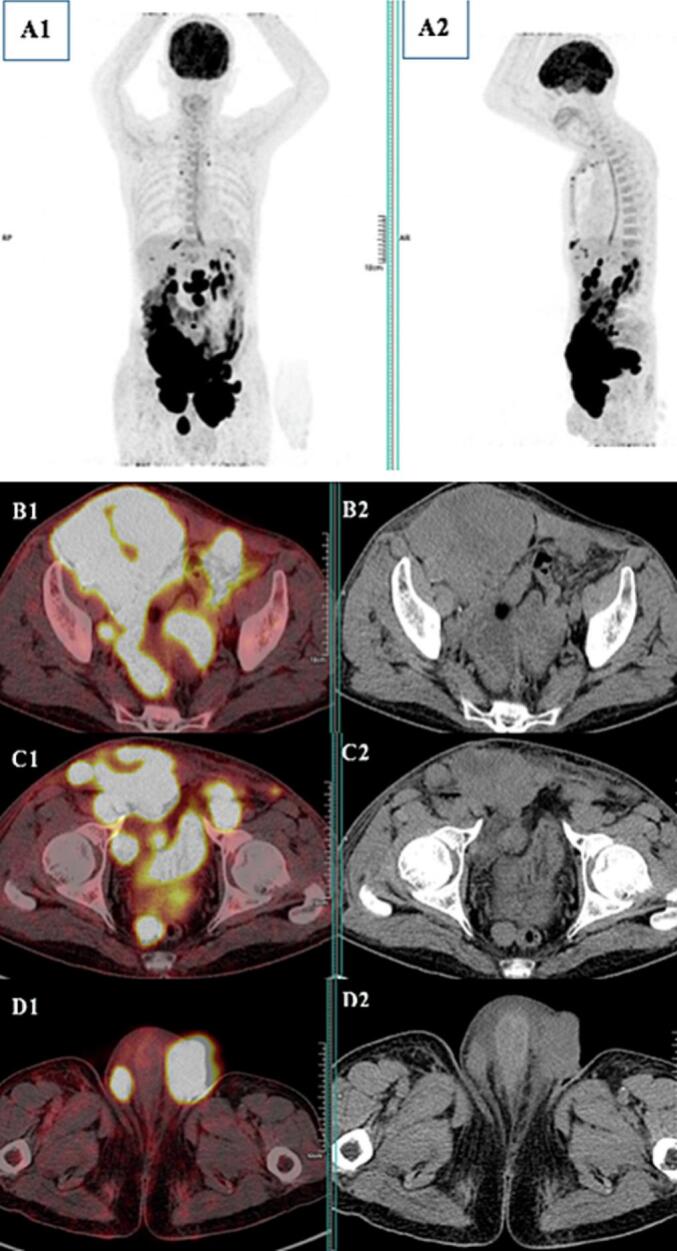
MIP image, face, and profile (A1 + 2). The axial slices of PET-CT (B1, C1, and D1) and CT (B2, C2, and D2) merger show diffuse tumor progression.

A 56-year-old patient with no previous pathological history was consulted for testicular pain that had been evolving for 15 days. He did not report systemic symptoms such as fever, night sweats, or weight loss during initial presentation. A testicular ultrasound showed the appearance of pre-suppurative right orchiepididymitis. He was treated with antibiotics and pain persisted, with the second follow-up ultrasound showing progressive right orchiepididymitis and a left testicular nodule in favor of pseudo-nodular orchitis. Granulomatous or brucellian involvement should be ruled out. Wright's serology came back negative, sperm culture negative: no pathogenic germ and absence of BAAR. The PCR in semen for *Mycobacterium tuberculosis* was negative. A third follow-up testicular ultrasound showed two testicles with suspicious ultrasound appearance (abnormal architecture and nodular appearance) and suspicious right inguinal adenomegaly. An abdominopelvic CT scan showed preaorticocaval and right iliac adenopathies, suggesting secondary testicular involvement. Although the imaging revealed suspicious lymphadenopathy, orchiectomy was prioritized for histopathological diagnosis, given the high suspicion of testicular malignancy and the worsening local symptoms. Although systemic involvement was suspected on CT, orchiectomy remains a standard diagnostic and therapeutic intervention in suspected testicular tumors. It allows rapid histological confirmation and can help reduce tumor burden prior to systemic therapy. Tumor marker assays were performed: AFP 15 IU/mL, LDH 266 IU/L, HCG < 16 ng/mL. The patient was admitted to the urology department for inguinal testicular exploration. Subsequently, a definitive systemic staging, including PET/CT, was performed after surgery. A right orchiectomy was performed with the following anapath findings: Aspect of diffuse large-cell B lymphoma that infiltrates all testicular structures: Reactive T lymphocytes and tumor cells negative for PLAP, C kit and ¾ OCT. Presence of perineural sheathing with circumferential margins of tumor resection. 18FDG PET-CT was requested as part of the initial disease extension study. It (Fig. 1, Fig. 2) showed involvement of the supradiaphragmatic and subdiaphragmatic lymph nodes with diffuse nodular involvement at the peritoneal level, as well as primary testicular involvement, probably bilateral. The patient received eight courses of R-CHOP chemotherapy, including: Rituximab, Cyclophosphamide, Hydroxydaunorubicin, vincristine sulfate (Oncovin) and Prednisone. He referred us for post-therapy PET-CT evaluation. It showed a good but partial metabolic response (Fig. 3) with disappearance of hypermetabolic supradiaphragmatic and testicular lymph node lesions, as well as most subdiaphragmatic and peritoneal lesions. However, two left inguinal lymph nodes and one abdominal parietal hypermetabolic lesion persisted (Deauville 5). Although the patient no longer adherent to scheduled chemotherapy sessions, he continued to be intermittently present for imaging studies and symptomatic evaluations. Upon investigation, it was revealed that nonadherence was due to personal factors, including financial hardship, limited family support, and emotional distress related to the prognosis. These factors contributed to discontinuity in the therapeutic process, despite the efforts of the medical team to re-engage him in treatment. The course of the disease was marked by progression, and the patient underwent a third PET-CT scan for suspected progression (Fig. 4), which revealed multiple hypermetabolisms of the supradiaphragmatic lymph nodes (prevascular, bilateral parasternal, bilateral anterior phrenic, inframediastian space right posterior) and subdiaphragmatic lymph nodes (gastric minor curvature, coeliomesenteric, primitive iliac, bilateral internal and external iliac), as well as a hypermetabolic right scrotal mass: Aspect in favor of diffuse tumor progression. The patient died a few weeks later.

## 3 Discussion

The typical presentation of LTP is unilateral, painless, progressive scrotal swelling, often accompanied by a hydrocele in an older man, with a median age of diagnosis of 67 years. The presence of systemic symptoms almost always indicates disseminated disease. Regional (paraaortic) lymph node involvement is present in 20–30 % of cases, and bilateral testicular involvement (usually subclinical) is present in around 10 % of cases, requiring specific investigation during imaging and staging examinations. Inguinal orchiectomy is the preferred diagnostic (and therapeutic) procedure, with consideration given to blind biopsy of the contralateral testis. Staging procedures should include routine imaging of the central nervous system (CNS), as well as cerebrospinal fluid (CSF) cytology and flow cytometry, given the propensity to CNS involvement, even when the disease appears clinically localized and low volume, and HIV serology [4]. >90 % of LTPs are diffuse large B-cell lymphomas (DLBCL), the remaining cases being Burkitt lymphomas, often in HIV+ patients, rare T-cell lymphomas, and the rare distinct syndrome of primary follicular lymphoma (FL), typically histological grade 3, lacking Bcl-2 expression and t translocation (14, 18), Historically, patients with LTP have often received inadequate systemic therapy due to a poor understanding of the aggressive natural history of the disease, the lack of significant curative potential of locoregional therapies used alone, and the propensity for widespread systemic dissemination and a very poor prognosis in the event of relapse. Large historical series from the prerituximab era describe a median survival of 12–24 months, with 5-year survival rates of 15–50 %, characterized by a temporal pattern of frequent relapses in the first two years, but with a persistent risk of relapses beyond 10 years, with involvement of the contralateral testes in up to 40 % of patients in the absence of prophylactic irradiation. Other distinctive patterns of relapse include the CNS, both leptomeningeal and parenchymal, in equal proportions in 20–35 % of cases in the absence of effective prophylaxis. Relapse systemic sites are often extranodal and involve soft tissues such as skin and lungs, and once manifested, relapsed disease has a very poor prognosis and is rarely curable [5]. This case also highlights a crucial issue in the treatment of aggressive malignancies: patient adherence. Despite a partial metabolic response, the patient discontinued chemotherapy prematurely. This may have been due to psychosocial, financial, or logistical barriers, illustrating the importance of multidisciplinary care and support systems in ensuring treatment continuity. In the absence of randomized trials, these observations underpin current treatment recommendations, derived from the prospective phase II IELSG10 study of 53 chemotherapy patients: six cycles of rituximab (R-CHOP), contralateral testicular irradiation, and prophylaxis of the CNS, ideally with intrathecal treatment, as well as systemic administration of high-dose antimetabolites, capable of penetrating the CNS parenchyma, such as cytarabine or methotrexate [6].

## 4 Conclusion

Primary testicular lymphoma (PTL) is a rare but aggressive malignancy that requires a timely diagnosis and appropriate treatment strategies. This case highlights the crucial role of FDG-PET/CT in the management of PTL, particularly in assessing the stage of disease staging and treatment response. FDG uptake reflects the metabolic activity of lymphoma cells, making it a valuable tool for identifying areas of involvement that may not be apparent in conventional imaging. Furthermore, the use of FDG-PET/CT facilitates early evaluation of therapeutic efficacy, guiding further management decisions and potentially improving patient outcomes. Continued research on the utility of FDG in PTL will improve our understanding and approach to this challenging condition, ultimately contributing to more personalized and effective treatment strategies.

## Consent

Written informed consent was obtained from the patient for the publication of this case report and accompanying images. A copy of the written consent is available for review by the Editor-in-Chief of this journal upon request.

## Ethical approval

Not applicable.

## Funding

This research did not receive specific grants from the public, commercial, or nonprofit sectors.

## Author contribution

All authors participate in the treatment of the patients, writing, and approving the manuscript.

## Guarantor

Mohamed Ali Chaouch.

## Research registration number

N/A.

## Conflict of interest statement

No conflict of interest to disclose.
